# Study on the Carbonation Behavior of Cement Mortar by Electrochemical Impedance Spectroscopy

**DOI:** 10.3390/ma7010218

**Published:** 2014-01-03

**Authors:** Biqin Dong, Qiwen Qiu, Jiaqi Xiang, Canjie Huang, Feng Xing, Ningxu Han

**Affiliations:** Guangdong Province Key Laboratory of Durability for Marine Civil Engineering, The Key Laboratory on Durability of Civil Engineering in Shenzhen, School of Civil Engineering, Shenzhen University, Shenzhen 518060, Guangdong, China; E-Mails: incise@szu.edu.cn (B.D.); qiuqiwen0422@163.com (Q.Q.); xjq517169100@gmail.com (J.X.); 18718575712@163.com (C.H.); xingf@szu.edu.cn (F.X.)

**Keywords:** electrochemical impedance spectroscopy, mortar, electrochemical model, parameter, carbonation depth, prediction

## Abstract

A new electrochemical model has been carefully established to explain the carbonation behavior of cement mortar, and the model has been validated by the experimental results. In fact, it is shown by this study that the electrochemical impedance behavior of mortars varies in the process of carbonation. With the cement/sand ratio reduced, the carbonation rate reveals more remarkable. The carbonation process can be quantitatively accessed by a parameter, which can be obtained by means of the electrochemical impedance spectroscopy (EIS)-based electrochemical model. It has been found that the parameter is a function of carbonation depth and of carbonation time. Thereby, prediction of carbonation depth can be achieved.

## 1. Introduction

Mortar has always found wide application in the construction industry, which plays a bonding, padding and protective role in concrete structures. Due to its high strength, low cost and convenient fabrication, mortars have been used as isolating lining materials in cisterns, wells, aqueducts, shafts and duct drains, as well as supporting materials for pavement sand mosaics, plasters on external and internal walls, supporting materials for frescoes, and as joint mortars of masonry structures [1]. The properties of mortar exert a great influence in concrete structure as mortar occupies a large amount of volume inside concrete. Much has been published in the literature: mortars are often affected by environmental degradation [2], causing a severe reduction of the durability of the reinforced concrete or masonry structure. Among all the aggressive elements (e.g., ions attack, freeze thawing), carbonation is the most common as well as the most harmful. In this sense, investigation of carbonation properties of mortars is of great value to durability design of concrete structure. Carbonation is actually a neutralization reaction. Reacting with the CO_2_ dissolved in the pore water, the highly alkaline components in concrete like Ca(OH)_2_, hydrated calcium silicate (C-S-H), *etc.* can be transformed into calcite crystals CaCO_3_ [3–9]. On the one hand, carbonation causes the reduction of pH value in the concrete pore solution, which leads to destroy the passivation oxide layer on the surface of the steel reinforcement [10–15]. On the other hand, contraction of concrete is very likely to be aggravated, giving rise to more cracks in concrete [16,17]. Therefore, carbonation has a considerable impact on durability of concrete structure [18–22].

One of the traditional ways of determining carbonation depth is to spray phenolphthalein indicator onto the surface of a split concrete prism [23]. Although the method retains its popularity nowadays, there are obvious disadvantages to this method. The biggest one is that the test must take samples out of the concrete structure, which indicates a destructive test. In addition, not easily detecting the pH value in a partially carbonated zone and vision illusion caused by darkly colored aggregate are also challenges [23]. Another negative side to be mentioned is that accuracy of measurement relies on the skill and experience of the person who performs the test.

As a sort of nondestructive testing, the electrochemical impedance spectroscopy (EIS) method is able to reflect the micro-structural changes in the cementitious materials under variously natural exposure environments, which has been viewed as a promising way to study the physical and chemical properties of cementitious materials [24–30].

The objective of this paper is firstly to obtain the electrochemical impedance data of the carbonated mortar then, to apply the electrochemical model to describe the carbonation behavior of mortar. Finally, obtaining the functional relationship between the fitted parameter of the model and the carbonation time, the prediction of carbonation depth is thought to be achieved.

## 2. Experimental Section

Cement: P.O 52.5 Portland cement, a product of the Starfish Onoda cement limited company of Shenzhen.

Water: normal tap water.

Sand: The standard sand derived from Xiamen ISO Stand Sand Company, China.

Mortar specimens with dimensions of 160 mm (length) × 40 mm (height) × 40 mm (thickness) were prepared with a cement/sand ratio of 1:2, 1:3 and 1:4 at the room temperature 20 °C as well as 95% of relative humidity. All specimens were water-cured for 28 days.

**Figure 1.**
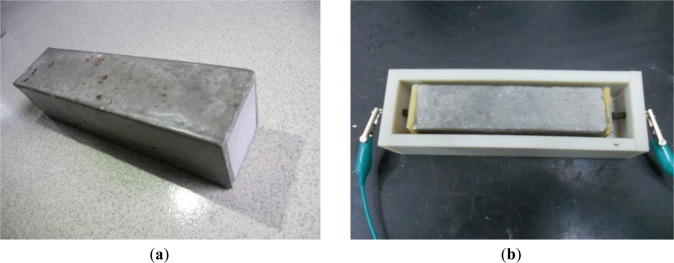
(**a**) The specimen; and (**b**) the mold for the EIS test.

Before exposing the mortars to accelerating carbonation, they were sealed with wax, with both ends of the specimens left open to carbonation (see Figure 1a). The carbonation is carried out in a carbonation accelerating chamber filled with 20% commercial CO_2_ concentration. Regarding temperature and humidity, the carbonation test is set as 29–31 °C and 65%–70%, respectively.

EIS measurement was carried out by Princeton Applied Research Co. (PAR, Oak Ridge, TN, USA) Potentiostat/Galvanostat 283 with a frequency range of 0.01 Hz–1 MHz. The test was performed at 0, 3, 7, 14, 28, 36, 60 days, respectively. The mold for electrochemical impedance measurement is shown in Figure 1b.

The carbonation depth was measured according to Chinese Standard (GBJ820-85) “*Standard for test methods of long-term performance and durability of ordinary concrete*” [31]. To start with, the test specimens were taken out of the carbonation chamber at given carbonation ages of 0, 3, 7, 14, 28, 36, 60, 90 and 120 days, and then split into two blocks with transverse exposed fresh surfaces. The next step was to cleaning and spraying the fresh surface with the phenolphthalein pH indicator (1% ethanol solution with 1g phenolphthalein and 90 mL 95.0% (V/V) ethanol diluted in water to 100 mL solution). In order to alleviate the experimental error, the carbonation depth was measured at 7 different points along the carbonation front. Measuring to nearest 0.1 mm with a digital caliper, the average depth value was calculated as the final experimental result.

## 3. Results and Discussion

**Figure 2.**
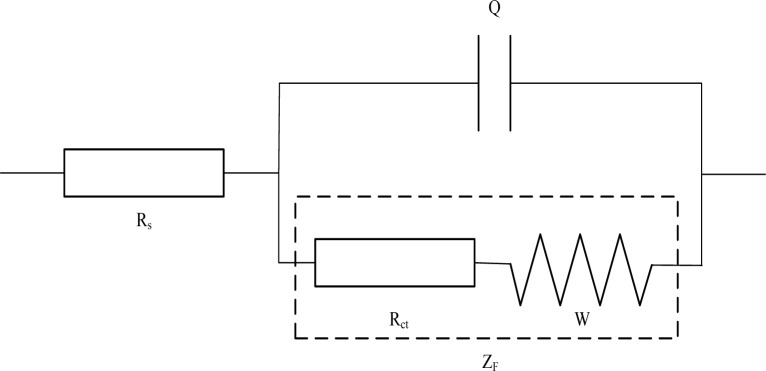
The Randles equivalent circuit for a general electrochemical system.

Generally, a simple electrochemical system can be simulated as a typical equivalent circuit shown in Figure 2, which can be described as *R*_s_(*Q*(*R*_ct_*W*)) by CDC (circuit description code).Where, *R*_s_ is the solution resistance, *Q* corresponds to the double-layer capacitance of the electrodes/electrolyte interface [32]. It should be emphasized that the value of *Q* is associated with CPE (constant phase element), which is generally attributed to distributed surface reactivity, surface inhomogeneity, roughness or fractal geometry, electrode porosity, and also to the current and potential distributions associated with electrode geometry. *Z*_F_ stands for the impedance of the Faraday’s procedure that occurs on the surface of the electrodes. Faraday’s procedure includes charge transfer procedure and charge diffusion procedure. As a result, Faraday impedance is represented by a serious connection of *R*_ct_ and *W*, in which *R*_ct_ stands for charge transfer resistance of the electrodes/electrolyte interface and *W* stands for Warburg resistance that caused by charge diffusion procedure.

As far as *R*_s_(*Q*(*R*_ct_*W*))’s concerned, electrolyte in this electrochemical system is regarded as a relatively stable component, only considering the electrochemical resistance (*R*_ct_). In this sense, other reactions are overlooked except the electrode reaction. However, the microstructure of cement-based materials is very complex [33], which includes air voids, capillary pores and gel pores [34]. These pores, always filled with solution, can be viewed as the spaces among solid phase like C-S-H gel and sand. The interfaces between the C-S-H gel and pore solution can have a great influence on the electrochemical behavior of cement-based materials [35]. Thereby, when it comes to study the electrochemical impedance of mortar materials, not only the reaction on the surface of the electrodes, but also the interaction between solid cement and liquid solution (solid/liquid double-phase) needs to be considered.

**Figure 3.**
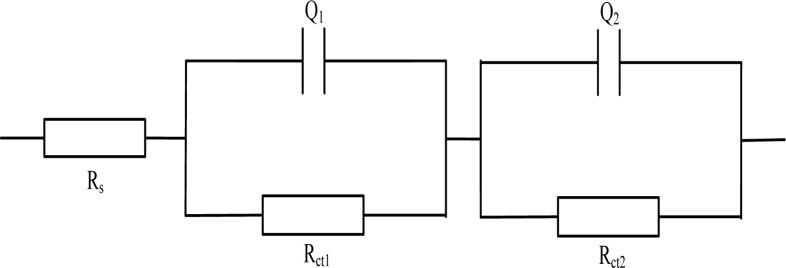
The simplified electrical equivalent circuit for hydration measurement of cement mortar.

In view of the mechanism, Gu Ping, *et al.* [36] put forward an electrical equivalent circuit model to study the impedance behavior of cement mortar, with roots in “solid-liquid interfaces” conceived. In view of the effect of the charge transfer and the outside testing electrode, the whole *R*_s_(*Q*_1_*R*_ct1_)(*Q*_2_*R*_ct2_) system (shown in Figure 3) was formed, where *R*_s_ represents the resistance of the electrolyte solution, *Q*_1_ corresponds to the double layer capacitance between the solid/liquid phases, *R*_ct1_ stands for the resistance caused by ion transfer procedure inside the cement mortar sample, *Q*_2_ stands for the double layer capacitance between cement mortar and electrodes; *R*_ct2_ stands for the resistance caused by the charge transfer procedure on the surface of the electrodes.

*T*he electrochemical model *R*_s_(*Q*_1_*R*_ct1_)(*Q*_2_*R*_ct2_) effectively characterizes the electrochemical impedance spectroscopy when the cement mortars are in dry condition. For dry specimens, as the pore solution in the materials is rather little and the ions scarcely diffuse on a large scale. It is therefore to say that the charge diffusion procedure impedance (Warburg impedance) is insignificant to mention in this case. However, as to the moist mortars (especially the ones that used in the neighborhood of seaside), the interior amount of solution is apparently higher, the Warburg impedance is supposed to be considered.

**Figure 4.**
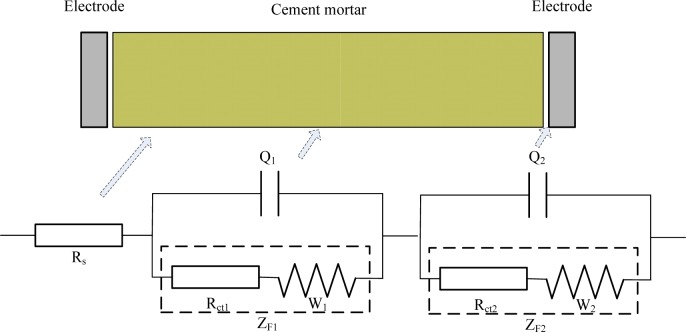
The equivalent circuit model proposed to investigate the cement mortar with carbonation process.

After consideration of the fact that ions in mortar would transfer on a large scale, a novel electrical circuit model is proposed for investigation of mortar’s carbonation (illustrated in Figure 4).

Where, *R*_s_ stands for the resistance of pore solution in mortar; *Q*_1_ stands for the double layer capacitance between the solid/liquid phases; *R*_ct1_ stands for the resistance caused by ions transfer procedure inside the mortar; *W*_1_ stands for Warburg resistance caused by ions diffusion procedure inside the mortar; *Q*_2_ stands for the double layer capacitance between mortar and electrodes; *R*_ct2_ stands for the resistance caused by the charge transfer procedure on the surface of the electrodes; *W*_2_ stands for Warburg resistance caused by the ion diffusion procedure on the surface of the electrodes. The CDC (Circuit Description Code) for this new equivalent circuit can then be described as *R*_s_(*Q*_1_(*R*_ct1_*W*_1_))(*Q*_2_(*R*_ct2_*W*_2_)), in which *R*_ct1_ + *W*_1_ = *Z*_F1_, standing for the Faraday impedance caused by the Faraday’s procedure inside the mortar; while *R*_ct2_ + *W*_2_ = *Z*_F2_, standing for the Faraday impedance caused by the Faraday’s procedure between the mortar and electrodes.

As concerning the mentioned-above equivalent electrical circuit model, the total impedance can be stated by the mathematical equation as below:

$$Z=R_{s}+\frac{Z_{F1}}{1+j\omega Z_{F1}Q_{1}}+\frac{Z_{F2}}{1+j\omega Z_{F2}Q_{2}}$$ (1)

$$Z=R_{s}+\frac{R_{\mathrm{ct}1}+\sigma_{1}\omega^{{-}_{2}^{1}}\left(1-j\right)}{1+j\omega R_{\mathrm{ct}1}Q_{1}+j\omega Q_{1}\left(\sigma_{1}\omega^{{-}_{2}^{1}}-j\sigma_{1}\omega^{{-}_{2}^{1}}\right)}+\frac{R_{\mathrm{ct}2}+\sigma_{2}\omega^{{-}_{2}^{1}}(1-j)}{1+j\omega R_{\mathrm{ct}2}Q_{2}+j\omega Q_{2}(\sigma_{2}\omega^{{-}_{2}^{1}}-j\sigma_{2}\omega^{{-}_{2}^{1}})}$$ (2)

The real part of *Z* is:

$$Z^{\prime }=R_{s}+\frac{R_{\mathrm{ct}1}+\sigma_{1}{\text{ω}}^{{-}_{2}^{1}}}{{\left(1+{\text{ω}}^{\frac{1}{2}}\sigma_{1}Q_{1}\right)}^{2}+{\text{ω}}^{2}Q_{1}^{2}{\left(R_{\mathrm{ct}1}+{\text{σ}}_{1}{\text{ω}}^{{-}_{2}^{1}}\right)}^{2}}+\frac{R_{\mathrm{ct}2}+{\text{σ}}_{2}{\text{ω}}^{{-}_{2}^{1}}}{{\left(1+{\text{ω}}^{\frac{1}{2}}{\text{σ}}_{2}Q_{2}\right)}^{2}+{\text{ω}}^{2}Q_{2}^{2}{\left(R_{\mathrm{ct}2}+{\text{σ}}_{2}{\text{ω}}^{{-}_{2}^{1}}\right)}^{2}}$$ (3)

And imaginary part of Z is:

$$Z^{\prime \prime }=j\left[\frac{\omega R_{\mathrm{ct}1}^{2}Q_{1}+2Q_{1}{\text{σ}}_{1}^{2}R_{\mathrm{ct}1}\omega^{\frac{1}{2}}+2\sigma_{1}^{2}Q_{1}+\sigma_{1}{\text{ω}}^{\frac{1}{2}}}{{\left(1+\omega^{\frac{1}{2}}\sigma_{1}Q_{1}\right)}^{2}+\omega^{2}Q_{1}^{2}{\left(R_{\mathrm{ct}1}+\sigma_{1}\omega^{{-}_{2}^{1}}\right)}^{2}}\right]+\frac{\omega R_{\mathrm{ct}2}^{2}Q_{2}+2Q_{2}\sigma_{2}^{2}R_{\mathrm{ct}2}\omega^{\frac{1}{2}}+2\sigma_{2}^{2}Q_{2}+{\text{σ}}_{2}\omega^{\frac{1}{2}}}{{\left(1+\omega^{\frac{1}{2}}\sigma_{2}Q_{2}\right)}^{2}+\omega^{2}Q_{2}^{2}{\left(R_{\mathrm{ct}2}+\sigma_{1}\omega^{{-}_{2}^{1}}\right)}^{2}}$$ (4)

where, σ_1_: the conductivity of cement mortar; σ_2_: the conductivity of electrodes; $\omega =2\pi f;\;W_{1}=\sigma_{1}\omega^{\left({-}_{2}^{1}\right)}(1-j);\;W_{2}=\sigma_{2}\omega^{\left({-}_{2}^{1}\right)}(1-j)$.

(1) When (ω→∞) (Very high frequency), that is, $\omega \gg {\left(\frac{\sigma }{R_{\mathrm{ct}}}\right)}^{2}$

$$Z^{\prime }=R_{s}+\frac{R_{\mathrm{ct}1}R_{\mathrm{ct}2}\left(Q_{1}+Q_{2}\right)\left(R_{\mathrm{ct}1}Q_{1}+R_{\mathrm{ct}2}Q_{2}-j\omega R_{\mathrm{ct}1}R_{\mathrm{ct}2}Q_{1}Q_{2}\right)}{{\left(R_{\mathrm{ct}1}Q_{1}+R_{\mathrm{ct}2}Q_{2}\right)}^{2}-{\left(j\text{ω}R_{\mathrm{ct}1}R_{\mathrm{ct}2}Q_{1}Q_{2}\right)}^{2}}$$ (5)

then

$$Z^{\prime }=R_{s}+\frac{R_{\mathrm{ct}1}R_{\mathrm{ct}2}\left(Q_{1}+Q_{2}\right)\left(R_{\mathrm{ct}1}Q_{1}+R_{\mathrm{ct}2}Q_{2}\right)}{{\left(R_{\mathrm{ct}1}Q_{1}+R_{\mathrm{ct}2}Q_{2}\right)}^{2}+{\left(\omega R_{\mathrm{ct}1}R_{\mathrm{ct}2}Q_{1}Q_{2}\right)}^{2}}$$ (6)

$$Z^{\prime \prime }=\frac{\omega {\left(R_{\mathrm{ct}1}R_{\mathrm{ct}2}\right)}^{2}\left(Q_{1}+Q_{2}\right)Q_{1}Q_{2}}{{\left(R_{\mathrm{ct}1}Q_{1}+R_{\mathrm{ct}2}Q_{2}\right)}^{2}+{\left(\omega R_{\mathrm{ct}1}R_{\mathrm{ct}2}Q_{1}Q_{2}\right)}^{2}}$$ (7)

Based on Equations (6) and (7), the following equation can be derived:

$${\left[Z^{\prime }-R_{s}-\frac{1}{2}\left(R_{\mathrm{ct}1}+R_{\mathrm{ct}2}\right)\right]}^{2}+{Z^{\prime \prime }}^{2}={\left(\frac{R_{\mathrm{ct}1}+R_{\mathrm{ct}2}}{2}\right)}^{2}$$ (8)

It is an equation standing for a half circle in the first quadrant.

When ω → 0 (Very low frequency);

$$Z^{\prime }=R_{s}+R_{\mathrm{ct}1}+\sigma_{1}\omega^{{-}_{2}^{1}}+R_{\mathrm{ct}2}+\sigma_{2}\omega^{{-}_{2}^{1}}$$ (9)

$$Z^{\prime \prime }=2\sigma_{1}^{2}Q_{1}+\sigma_{1}\omega^{{-}_{2}^{1}}+2\sigma_{2}^{2}Q_{2}+\sigma_{2}\omega^{{-}_{2}^{1}}$$ (10)

Based on Equations (9) and (10), the following equation can be derived:

$$Z^{\prime }=Z^{\prime \prime }-2\sigma_{1}^{2}Q_{1}-2\sigma_{2}^{2}Q_{2}+R_{s}+R_{\mathrm{ct}1}+R_{\mathrm{ct}2}$$ (11)

This is a linear equation.

**Figure 5.**
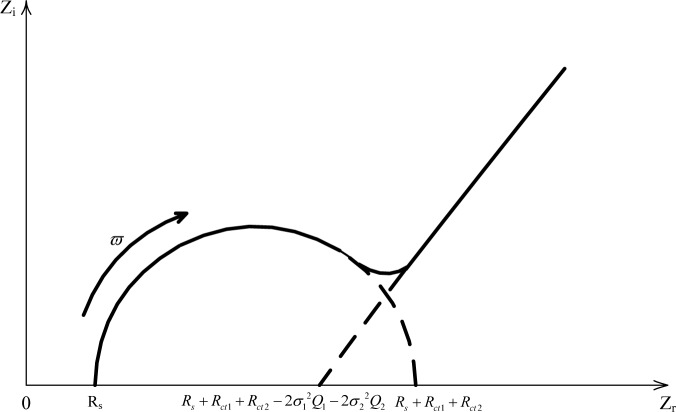
Curve on the complex plane corresponding to carbonation process of mortar.

Based on the derivation in Equations (8) and (11), a typical Nyquist curve of the electrochemical impedance spectroscopy for carbonation procedure in mortar can be drawn as Figure 5.

**Figure 6.**
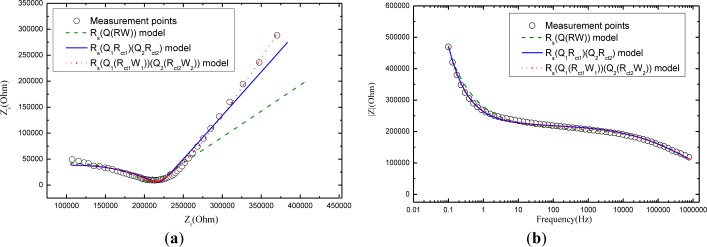

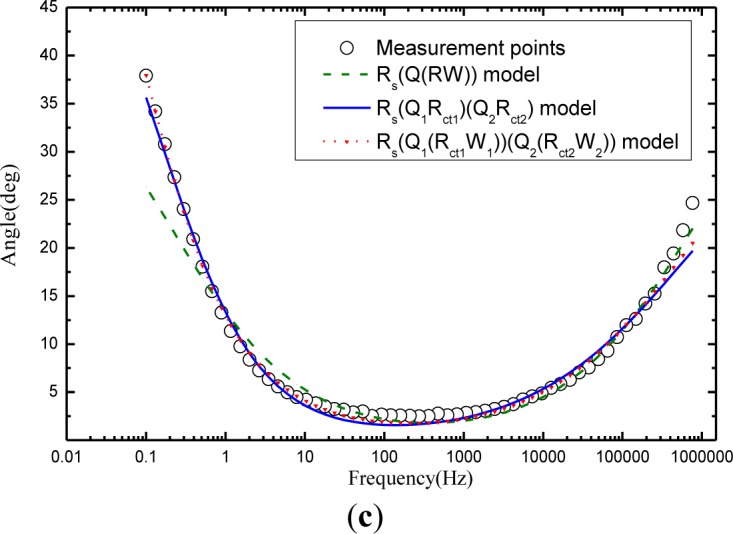
(**a**) The Nyquist plots; (**b**) Bode plots; and (**c**) Phase angle plots of the electrochemical impedance measurement for mortar with 1:3 cement/sand ratio at 21 day carbonation. The experiment data are shown as open circles. The dotted line is the fitting results based on *R*_s_(*Q*(*R*_ct_*W*)) model and the solid line is the fitting results based on *R*_s_(*Q*_1_*R*_ct1_)(*Q*_2_*R*_ct2_) model, together with the dots which represents the model *R*_s_(*Q*_1_(*R*_ct1_*W*_1_))(*Q*_2_(*R*_ct2_*W*_2_)).

Figure 6 compares the fitting results of electrochemical impedance measurement among the Randles model *R*_s_(*Q*(*R*_ct_*W*)), the model *R*_s_(*Q*_1_*R*_ct1_)(*Q*_2_*R*_ct2_), and the model *R*_s_(*Q*_1_(*R*_ct1_*W*_1_))(*Q*_2_(*R*_ct2_*W*_2_)) in the Nyquist figure. It can see clearly that model *R*_s_(*Q*_1_(*R*_ct1_*W*_1_))(*Q*_2_(*R*_ct2_*W*_2_)) that considers the charge diffusion procedure impedance (Warburg) in mortar performs the best fit for the impedance spectroscopy data while the Randles model *R*_s_(*Q*(*R*_ct_*W*)) shows a poor fit, demonstrating that the traditional model *R*_s_(*Q*(*R*_ct_*W*)) is not probable to study the carbonation mechanism of mortar. Although the more comprehensive model *R*_s_(*Q*_1_*R*_ct1_)(*Q*_2_*R*_ct2_) makes a good figure, it is inferior to the model *R*_s_(*Q*_1_(*R*_ct1_*W*_1_))(*Q*_2_(*R*_ct2_*W*_2_)). The phenomenon is not hard to explain: The samples in the whole experimental work are wettish (cured for 28 days in water and then kept in carbonation chamber of 95% relative humidity). As noted above, Warburg impedance is supposed to be taken into account for those of mortars in wet state. But model *R*_s_(*Q*_1_*R*_ct1_)(*Q*_2_*R*_ct2_)leaves out the Warburg impedance of the mortar. If the mortar turn dry, the most comprehensive model *R*_s_(*Q*_1_(*R*_ct1_*W*_1_))(*Q*_2_(*R*_ct2_*W*_2_)) will convert to electrical equivalent circuit of *R*_s_(*Q*_1_*R*_ct1_)(*Q*_2_*R*_ct2_) as the “*W*_1_” and “*W*_2_” are cleared away.

In the light of the fitting results above, conclusion can naturally be drawn that the new model *R*_s_(*Q*_1_(*R*_ct1_*W*_1_))(*Q*_2_(*R*_ct2_*W*_2_)) proposed in this paper is able to explore the properties of carbonation of mortar.

**Figure 7.**
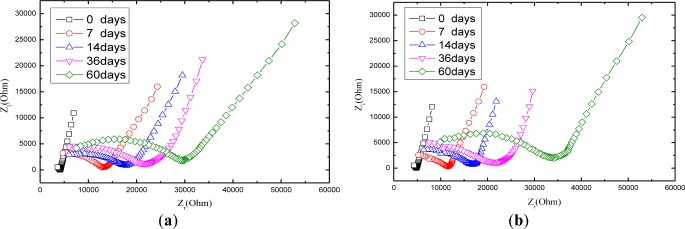

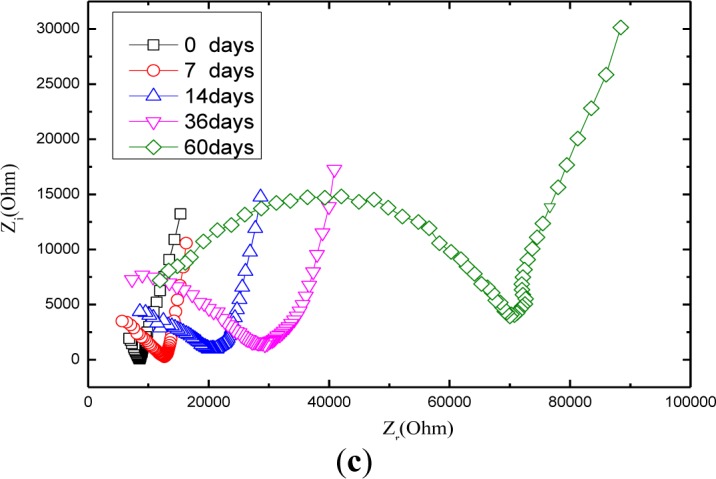
The Nyquist curves measured at different carbonation time for the mortar: (**a**) mortar with 1:2 cement/sand ratio; (**b**) mortar with 1:3 cement/sand ratio; (**c**) mortar with 1:4 cement/sand ratio.

Figure 7 shows the Nyquist curve of mortars with carbonation time. It is obvious that the radius of the half circle of Nyquist curve increases while the carbonation time extends, which is attributed partially to that CO_2_ dissolved in the electrolyte solution reacts with OH^−^ ions generated by the hydration of the cement so that the concentration of OH^−^ ions tends to decline. Among all the ions in the cement-based materials, OH^−^ ion is thought of as the most conductive [37]. In the light of what has been mentioned above, carbonation can lead to increase the resistance of charge transfer. Another factor that contributes to increase the Nyquist arc is that calcium carbonate has a very low solubility and precipitates inside the concrete pores, reducing the porosity and increasing the density of mortar [38,39], which consequently tends to rises the resistance of the ions transfer process.

**Figure 8.**
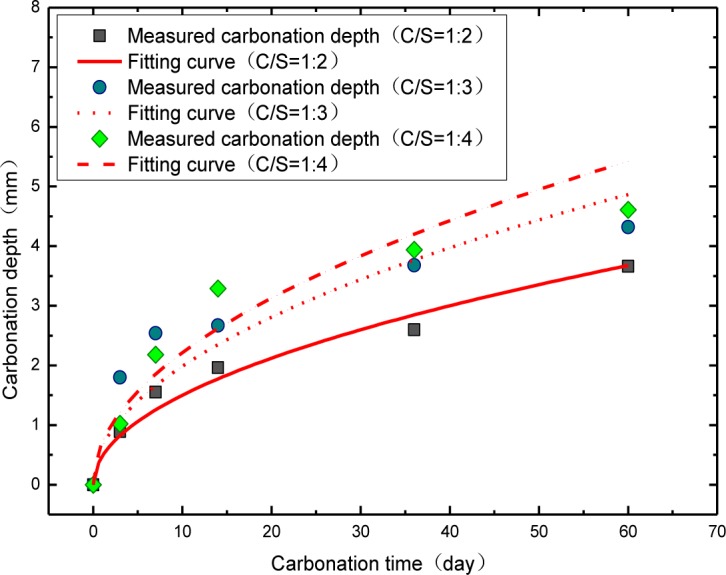
The experimental result of carbonation depth for mortars with different cement/sand ratio (1:2,1:3,1:4) and its fitting result.

With the purpose to investigate the quantized links between the resistance caused by ion transfer procedure inside the mortar sample (*R*_ct1_) and carbonation depth, the fitting parameter *R*_ct1_ of *R*_s_(*Q*_1_(*R*_ct1_*W*_1_))(*Q*_2_(*R*_ct2_*W*_2_)) model at different carbonation time is listed in Table 1. Through the results obtained, we notice that *R*_ct1_ is an increasingly linear proportion to the carbonation time. In order to explore the quantitatively functional correlation between *R*_ct1_ and carbonation depth, the test of the carbonation depth for the mortar is carried out with the same carbonation cycle. The results shown in Table 2 and Figure 8 claim that the link between the carbonation depth and carbonation time is in line with the square-root-t-law, that is:

$$D=k\cdot \sqrt{t}$$ (12)

where, *D* is the carbonation depth; *k* is the carbonation coefficient, determined by the material properties.

The experimental results are in perfect agreement with other researchers [40–45].

Given that *R*_ct1_ value is linear with time while carbonation depth (*D*) follows the square-root-t-law, strong relations exist between carbonation depth (*D*) and *R*_ct1_ value:

$$D\sim \sqrt{R_{\mathrm{ct}1}}$$ (13)

**Figure 9.**
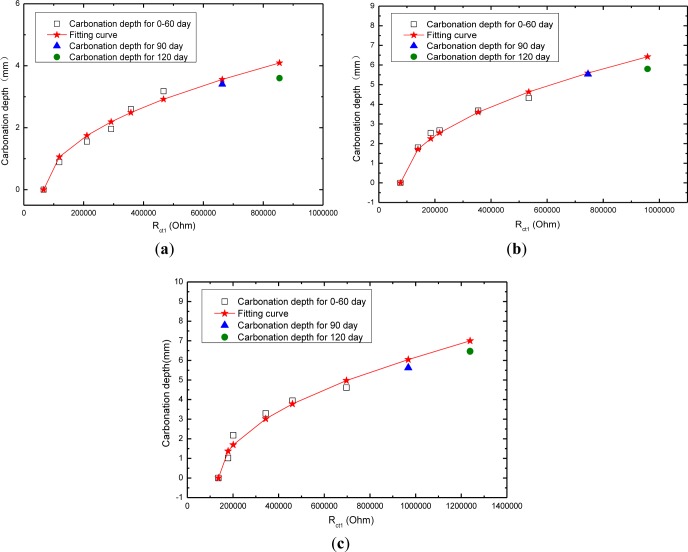
Comparison of the experimental results with fitting results based on for the mortar: (**a**) mortar with 1:2 cement/sand ratio; (**b**) mortar with 1:3 cement/sand ratio; (**c**) mortar with 1:4 cement/sand ratio.

A comparison between the experimental values and the estimated values of the carbonation depth both at 90 days and 120 days is shown in Figure 9 as well as Table 3. In Table 3, a calculated result of carbonation depth (C/S = 1:2) is estimated to reach 3.56 mm, while the measured carbonation depth is 3.40 mm. The relative error (%) between the experimental result and the prediction is only 4.71%. As to mortar of cement/sand ratio = 1:3, the carbonation depth is predicted as 5.59 mm, while the measured value is 5.53 mm, which claims the error of 1.08%. The 120 d carbonation depth also shows a good prediction. It demonstrates that electrochemical impedance spectroscopy fitting parameter *R*_ct1_ based on *R*_s_(*Q*_1_(*R*_ct1_*W*_1_))(*Q*_2_(*R*_ct2_*W*_2_)) model can be used to predict the cement carbonation depth in an acceptable manner.

## 4. Conclusions

Based on the test and the analytical results, the following conclusions could be drawn:

- It is of theoretical and practical significance to characterize the carbonation behavior and to predict the carbonation depth of mortar by EIS measurement. This approach can overcome the inherent constraints of phenolphthalein solution test.
- A novel equivalent circuit model with *R*_s_(*Q*_1_(*R*_ct1_*W*_1_))(*Q*_2_(*R*_ct2_*W*_2_)) has been proposed to provide detailed insight on the carbonation behavior of mortar, taking into account both the solid/liquid double-phase interaction and the Warburg impedance. The curve fitting results based on the proposed model show a high consistency with the experimental results.
- *R*_ct1_ value obtained by fitting calculation of the impedance data with the new electric circuit can be applied to characterize the carbonation behaviors of the mortar. Comparing with the experimental results and with modeling parameters, it is found that carbonation depth is the function of *R*_ct1_: $D\sim \sqrt{R_{\mathrm{ct}1}}$. This relationship can be utilized to predict the carbonation depth precisely. Experimental results demonstrate that the prediction is ≤11% error for most of the specimens.

## Figures and Tables

**Table 1.** The fitting result of *R*_ct1_ based on *R*_s_(*Q*_1_(*R*_ct1_*W*_1_))(*Q*_2_(*R*_ct2_*W*_2_)) model.

Carbonation time (day)	*R*_ct1_ value calculated from *R*_s_(*Q*_1_(*R*_ct1_*W*_1_))(*Q*_2_(*R*_ct2_*W*_2_)) model (Ohm)		
	*C*/*S* = 1:2	*C*/*S* = 1:3	*C*/*S* = 1:4
0	66,200	77,330	136,200
3	118,900	139,800	178,600
7	211,000	185,100	201,300
14	292,000	216,600	343,300
36	358,000	354,500	460,600
60	467,000	534,600	697,300
90	663,200	745,800	968,100
120	854,600	957,800	1,238,900

**Table 2.** The average carbonation depth for mortars with different cement/sand ratio (1:2,1:3,1:4).

Carbonation time (day)	Carbonation depth (mm)		
	C/S = 1:2	C/S = 1:3	C/S = 1:4
0	0.00	0.00	0.00
3	0.89	1.80	1.02
7	1.55	2.54	2.18
14	1.96	2.67	3.29
36	2.60	3.68	3.94
60	3.18	4.32	4.61
90	3.40	5.53	5.62
120	3.60	5.80	6.46

**Table 3.** The comparison of average measured carbonation depth for mortars with different cement/sand ratio.

Carbonation Depth		Cement/Sand Ratio		
		1:2	1:3	1:4
90 day	Measured carbonation depth (mm**)**	3.40	5.53	5.62
	Calculated value (mm)	3.56	5.59	6.04
	Variation (%)	4.71	1.08	7.47

120 day	Measured carbonation depth (mm)	3.60	5.80	6.46
	Calculated value (mm)	4.09	6.42	7.00
	Variation (%)	13.61	10.69	8.36
